# The role of microglia in the pathogenesis of diabetic-associated cognitive dysfunction

**DOI:** 10.3389/fendo.2023.1246979

**Published:** 2024-01-11

**Authors:** Wenwen Xu, Xinyu Wang, Xunyao Hou, Yan Yang, Rongrong Ma, Renjun Lv, Qingqing Yin

**Affiliations:** ^1^ Department of Ophthalmology, Shandong Provincial Hospital Affiliated to Shandong First Medical University, Jinan, Shandong, China; ^2^ Department of Geriatrics, Shandong Provincial Hospital Affiliated to Shandong First Medical University, Jinan, Shandong, China; ^3^ First Clinical Medical College, Shandong University of Traditional Chinese Medicine, Jinan, China; ^4^ Key Laboratory of Endocrine Glucose & Lipids Metabolism and Brain Aging, Ministry of Education, Department of Geriatric Neurology, Shandong Provincial Hospital Affiliated to Shandong First Medical University, Jinan, Shandong, China

**Keywords:** diabetic-associated cognitive dysfunction, pathogenesis, microglia, neuroinflammation, type 2 diabetes mellitus

Diabetic-associated cognitive dysfunction (DACD) is a major central nervous system (CNS) complication in patients with diabetes and is attracting increasing attention (1). Cognitive dysfunction in patients with diabetes includes decreased learning ability, memory, problem-solving, attention and information processing speed (2). Microglia, as immune cells of the central nervous system, play an important role in maintaining tissue homeostasis and contribute to brain development under normal conditions (3). When brain damage occurs, microglia are activated to secrete either proinflammatory factors that increase cytotoxicity or anti-inflammatory neuroprotective factors that aid wound healing and tissue repair. However, excessive microglial activation damages the surrounding normal neurons, and the factors secreted by the dead or dying neurons in turn exacerbate the chronic activation of microglia, causing progressive loss of neurons, accelerating the progression of DACD. Hence, clarifying the role of microglia in DACD and resolving chronic inflammation mediated by microglia is helpful to bear a novel treatment strategy for DACD.

Mature microglia in the postnatal brain respond to their extracellular environment rapidly through a wide variety of surface molecules, including cytokines, chemokines, purines, hormones, and neurotransmitters (4). Microglia express common markers, similar to other macrophages residing in tissues, such as the fractalkine receptor CX3CR1, CSF1R, the integrin CD11b, surface glycoproteins F4/80 and CD68, ionized calcium-binding adaptor molecule 1 (Iba1), and pan-hematopoietic CD45. Microglia in the central nervous system injury and disease has a complicated connection, referring to “activation”. Microglial activation has been detected by positron emission tomography in patients with mild cognitive impairment. M1 phenotype is the classical activation, be considered pro-inflammatory and neurotoxic, as well as closely related to the concept of “activated” microglia. M2 phenotype or alternative activation, be considered anti-inflammatory and neuroprotective, plays an important role in resolving inflammation, clearing toxicity, and preserving brain tissue (5). These cells can be further divided into subgroups, each with its unique function in the CNS. M2a is involved by removing cellular debris and regeneration, M2b in immune regulation, and M2c in neuroprotective and anti-inflammatory cytokine production such as interleukin-10 (IL-10) and interleukin-4 (IL-4) (6). However, this dualistic classification of good or bad microglia is inconsistent with the wide repertoire of microglial states and functions in development, plasticity, aging, and diseases that were elucidated in recent years. Macrophage responses are more complex than simply “M1” and “M2”. In the case of microglia, the advent of single-cell technologies provided clear evidence that microglia in the living brain do not polarize to either of these categories, often co-expressing M1 and M2 markers. Microglia activation was more diverse and dynamic than previously anticipated, both in terms of omics features and functional outcomes, suggesting that microglia respond differently to different diseases. The altered phenotype allows microglia to exert a protective role, which is regulated by the brain microenvironment. When organotypic brain slices from aged APP/PS1 mice were co-cultured with young neonatal WT mice, aged microglia from aged mice moved towards amyloid plaques and cleared plaque halos (7). Opsonizing mediators from young microglia also promoted the proliferation and phagocytosis of amyloid plaques by old microglia, suggesting that microglia function could be reversed by microenvironment-driven therapies.

Insulin resistance is the pathophysiological basis and the core mechanism of DACD (8). In the early stage of DACD, insulin resistance and neuroinflammation are interrelated pathological features, and they are considered to be the two main factors that directly or indirectly lead to synaptic destruction and neurophysiological alterations (9). Brain insulin resistance occurs through the release of pro-inflammatory cytokines, peripherally produced pro-inflammatory cytokines such as tumor necrosis factor-α (TNF-α), interleukin-6 (IL-6), interleukin -12 (IL-12) and interleukin-1β (IL-1β) can cross the blood-brain barrier, leading to neuroinflammation and central insulin resistance (10). It is noteworthy that IL-1β and IL-6 play a particularly important role in microglia function and IL-1β signaling pathways enhance the pro-inflammatory response of microglia (11). Occurrence and development of DACD, the insulin signaling pathway is impaired in the brain and insulin function adjustment by potential glucoregulatory function in the CNS neurons (12). It is becoming increasingly apparent that the insulin level is increased, the degradation of the insulin-degrading enzyme, interfering with amyloid plaques clearance and disrupting neurons. The severity of the microglial response depends on the severity of the nerve injury.

As resident immune cells of the CNS, microglia are the main effector cells of neuroinflammation and latently adjust their inflammatory processes (13). Most of the histological features of many neurological diseases are characterized by “microgliosis”, including changes in microglia morphology, changes in gene expression, migration, growth, and proliferation after injury. Multiple studies have demonstrated that factors such as neuronal apoptosis, oxidative stress, neuroinflammation and altered neurogenesis may play a role in DACD (14, 15). Neuroinflammation is the protection and repair of tissue damage and extreme deviations from internal balance in the central nervous system. It is not appropriate to always assume that neuroinflammation is harmful. Instead, it should be recognized that each inflammatory response may have adaptive or maladaptive effects, depending on the context. There is many clinical evidence of cerebral inflammation in type 2 diabetes mellitus (T2DM) and experimental findings support the notion that microglial activation contributes to neuronal injury and cognitive impairment in T2DM models (16). DACD patients and mouse models have elevated levels of inflammatory markers, and several DACD risk genes associated with innate immune function have been identified, suggesting that neuroinflammation plays a critical role in DACD pathogenesis (14, 17). APP/PS1xdb/db mice show significant increases in microglial activation (18). Our previous studies suggested that changes in the abundance of hippocampal cell populations, most notably microglial cell populations, which confirm the novel opinion that microglia play a vital role in DACD (19). Research showed that disease-associated microglia (DAM), the subpopulation of microglia, could play a neuroprotective role to alleviate the disease by enhancing phagocytosis in the late stages of AD (20). Given that DACD as the early stage of dementia, it may be relevant to cerebrovascular damage. The pro-inflammatory DAM emerges in hippocampus of T2DM mouse model and are characterized by expression of pro-inflammatory genes and regulators (21). Therefore, some studies have improved DACD by reducing microglia-mediated neuroinflammation.

Microglia cell membrane expression is rich in receptors in response to changes in “danger” signals in the surrounding environment (22). Different types of transcription factors can tightly control the phenotypic diversity and function of microglia (23). The markers of tissue damage caused by persistent cerebral ischemia and hypoxia are related to the expression of Toll-like receptor 4 (TLR-4) on the membrane of microglia. TLR-4 and triggering receptor expressed on myeloid cells-2 (TREM-2) to promote the activation of microglia (24, 25). TREM-2 is mostly involved in aiding microglial phagocytosis and its variants found in AD patients most likely impair this microglial function (26).

Activated microglia move to the injury site, phagocytoses apoptotic neurons and cell debris, and produces a large number of pro-inflammatory mediators, resulting in a persistent inflammatory microenvironment in the brain, which further causes the death of neurons and neural progenitor cells, leading to a vicious cycle characterized by microglial activation, inflammatory factor release, and neuronal death. The major signaling pathways that drive microglia to transform into a proinflammatory involve the MAPK pathway, JAK/STAT pathway and PI3K/Akt pathway which mainly activates the transcription factor NF-κB leading to the synthesis of various proinflammatory cytokines. In the contrast, metabolic receptors involved in lipid metabolism, and Sirt1-mTORC1 signaling mostly function to downregulate or inhibit the proinflammatory pathways (27). Therefore, targeting microglial signaling pathways needs to be studied as the foundation of therapy target DACD.

Microglia function does not occur in isolation but is linked to the activity of neurons, astrocytes, and vascular cells. Neurons exhibit hyperactivity in response to neurotoxic factors, hyperglycemia, and hyperlipidemia, and secrete latent microglial activators such as matrix metalloproteinase-9, ATP, and chemokine. Meanwhile, p38 MAPK activation in microglia results in the production of mediators such as neurotrophins and inflammatory factors to regulate synaptic transmission and inflammation, respectively. Thereby, targeting microglial signaling through inhibiting the actions of ATP receptors, matrix metalloproteinase-9, chemokines, p38 MAPK, and IL-6, TNF-α, and IL-1β may contribute to new therapies for DACD. Meanwhile, the tendency of microglia concentrated to neuronal axons and dendrites play a pivotal role between neurons and microglia interactions. Delayed activation of microglia may contribute to endocrine dysregulation and increased sympathetic nerve activity in diabetic rats (28). Overexpression of a dominant negative mutant of the transcription factor cAMP response element-binding protein in neurons induces neuronal apoptosis and microglial activation (29).

As macrophages of the brain parenchyma, microglia are involved in many key central nervous system functions, from gliosis, vascularity and neurogenesis to synapsis and myelination, through their motor processes, and release of soluble factors. Microglia plays an important role in synapse formation, pruning, and eliminating and regulating synaptic function. In the process of normal brain development, elimination includes removing unnecessary excitatory and inhibitory synapses synaptic connections, which is crucial for the formation of mature and effective neural circuits. Microglia can improve synaptic plasticity through the expression and release of brain-derived neurotrophic factor (BDNF), secrete cytokines to regulate synaptic plasticity, such as TNF-α. However, microglia near amyloid plaques were reduced in phagocytosis in genetic AD mice with concomitant T2DM (30). Therefore, excessive phagocytosis of microglia leads to neuronal synaptic dysfunction and aggravates the progress of cognitive function.

With the deepening understanding of the pathological progress underlying DACD, microglia play an increasingly prominent role in the brain microenvironment. Microglia have a variety of functions that are highly dynamic as well as interacting with many types of cells in response to environmental changes or stimuli. At present, some therapies targeting microglia have been proposed in animal models, but their efficacy still needs to be validated in clinical trials (31, 32). Using lipid nanoparticles to deliver anti-inflammatory siRNA reduced neuroinflammation in a neurodegeneration model, highlighting the potential of lipid nanoparticles as therapeutic tools (33). In addition, there is growing interest in non-drug therapies as a promising intervention for the treatment of cognitive decline in DACD. Some rodent studies have shown that lifestyle changes, such as physical exercise and diet control, can also prevent microglia activation, reduce neuroinflammation, and improve cognitive function (34).

## Author contributions 

All authors listed have made a substantial, direct, and intellectual contribution to the work and approved it for publication.
